# Dissociative electron attachment to coordination complexes of chromium: chromium(0) hexacarbonyl and benzene-chromium(0) tricarbonyl

**DOI:** 10.3762/bjnano.8.225

**Published:** 2017-10-30

**Authors:** Janina Kopyra, Paulina Maciejewska, Jelena Maljković

**Affiliations:** 1 Faculty of Sciences, Siedlce University, 3 Maja 54, 08-110 Siedlce, Poland; 2 Laboratory for Atomic Collision Processes, Institute of Physics Belgrade, University of Belgrade, Pregrevica 118, 11080 Belgrade, Serbia

**Keywords:** benzene-chromium(0) tricarbonyl, chromium(0) hexacarbonyl, dissociative electron attachment, gas phase reactions, mass spectrometry

## Abstract

Here we report the results of dissociative electron attachment (DEA) to gas-phase chromium(0) hexacarbonyl (Cr(CO)_6_) and benzene-chromium(0) tricarbonyl ((η^6^-C_6_H_6_)Cr(CO)_3_) in the energy range of 0–12 eV. Measurements have been performed utilizing an electron-molecular crossed beam setup. It was found that DEA to Cr(CO)_6_ results (under the given experimental conditions) in the formation of three fragment anions, namely [Cr(CO)_5_]^−^, [Cr(CO)_4_]^−^, and [Cr(CO)_3_]^−^. The predominant reaction channel is the formation of [Cr(CO)_5_]^−^ due to the loss of one CO ligand from the transient negative ion. The [Cr(CO)_5_]^−^ channel is visible via two overlapping resonant structures appearing in the energy range below 1.5 eV with a dominant structure peaking at around 0 eV. The peak maxima of the fragments generated by the loss of two or three CO ligands are blue-shifted and the most intense peaks within the ion yield curves appear at 1.4 eV and 4.7 eV, respectively. (η^6^-C_6_H_6_)Cr(CO)_3_ shows a very rich fragmentation pattern with decomposition leading to the formation of seven fragment anions. Three of them are generated from the cleavage of one, two or three CO ligand(s). The energy of the peak maxima of the [(C_6_H_6_)Cr(CO)_2_]^–^, [(C_6_H_6_)Cr(CO)]^–^, and [(C_6_H_6_)Cr]^−^ fragments is shifted towards higher energy with respect to the position of the respective fragments generated from Cr(CO)_6_. This phenomenon is most likely caused by the fact that chromium–carbonyl bonds are stronger in the heteroleptic complex (η^6^-C_6_H_6_)Cr(CO)_3_ than in homoleptic Cr(CO)_6_. Besides, we have observed the formation of anions due to the loss of C_6_H_6_ and one or more CO units. Finally, we found that Cr^−^, when stripped of all ligands, is generated through a high-energy resonance, peaking at 8 eV.

## Introduction

Organometallic compounds are a large class of compounds with numerous applications such as homogeneous catalysts for the synthesis of fine chemicals or even enantiomerically pure products used in the pharmaceutical industry [1–3]. However, they also play an important role in nanotechnology. In fact, a number of organometallic complexes, originally designed for chemical vapor deposition (CVD) purposes, have also been recognized as promising precursors for focused electron beam induced deposition (FEBID), a process to fabricate three-dimensional metal-containing nanoscale structures [4–5]. FEBID is a direct-write technique in which a highly focused, high-energy electron beam impinges on precursor molecules physisorbed onto a substrate, thereby causing their dissociation, and in the ideal case, leading to pure deposit formation. However, the primary electron (PE) beam striking the substrate gives rise to a large amount of back-scattered electrons (BSEs) and secondary electrons (SEs) [6–8]. It is nowadays very well known that these low energy electrons (<100 eV) may induce fragmentation of the adsorbed precursor molecules via various decomposition processes such as dissociative ionization (DI), dipolar dissociation (DD), neutral dissociation (ND), and dissociative electron attachment (DEA) [8]. These reactions occur with relatively high cross sections and typically result in partial fragmentation of the precursor molecules [9–11]. Therefore, SEs may play a role in determining the composition of the FEBID deposits. Moreover, they may also be responsible for the broadening of the deposits beyond the width of the PE beam since secondary electrons create an electron flux beyond the focal area diameter of the primary beam.

To date there have been several papers devoted to the studies of the interaction of low energy electrons with gas-phase organometallic complexes. Particular attention has been paid to the compounds containing monodentate (e.g., carbonyl [12–14], trifluorophosphine [11,15], chloride [16]), bidentate (e.g., hexafluoroacetylacetone [17]), and mixed ligands (e.g., nitrosyl and carbonyl [9–10], methyl and methylcyclopentadienyl [18], π-allyl, carbonyl, bromide [19]). These studies cover both the fragmentation patterns and kinetics of electron attachment processes. It appears that for carbonyl compounds such as Ni(CO)_4_, Fe(CO)_5_, W(CO)_6_, Mo(CO)_6_, and Cr(CO)_6_ the rate constants at thermal energy are extremely high and range from 1–3 × 10^−7^ cm^3^·molecule^−1^·s^−1^ [13]. These values approach the maximum (s-wave) thermal attachment rate constant of 5 × 10^−7^ cm^3^·molecule^−1^·s^−1^ at a temperature of 298 K [20].

**Figure 1:**
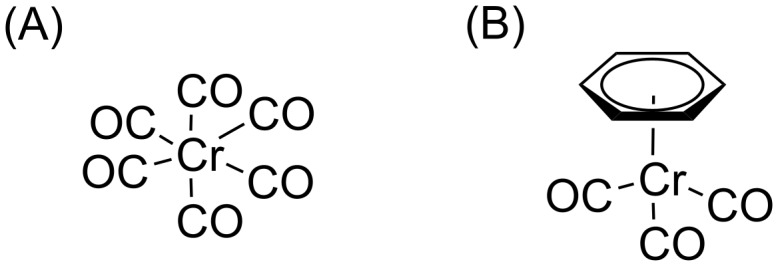
Structure of (A) chromium(0) hexacarbonyl and (B) benzene-chromium(0) tricarbonyl.

There is, however, still a need to find more efficient FEBIP precursors that will readily detach ligands upon interaction with electrons. According to the current understanding, precursors with large organic ligands are particularly unfavorable for FEBID because they lead to codeposition of large amounts of carbon [4,21]. However, as shown in this paper, for the case of a chromium complex carrying a benzene ligand, such large organic entities may be more easy to remove by electron irradiation than generally anticipated. Chromium complexes are of interest because they are used for various technological applications [22]. For instance, Cr is used in photomasks so that Cr-containing FEBID precursors are of interest for mask repair [23] and Cr(CO)_6_ has in fact been studied as a FEBID precursor earlier [24]. In the present work, we report the results from DEA to the gas-phase chromium(0) hexacarbonyl (Cr(CO)_6_) and benzene-chromium(0) tricarbonyl ((η^6^-C_6_H_6_)Cr(CO)_3_) [25] in the energy range of 0–12 eV. The first compound is homoleptic and hence contains one type of ligand, namely CO. The other compound is heteroleptic and contains both CO and a C_6_H_6_ ligand. CO is a monodentate ligand which means that only one atom within the ligand binds to the central metal atom. C_6_H_6_ is a η^6^ (hexahapto) ligand which corresponds to a contiguous series of six atoms that coordinate to the metal center. The molecular structure of both complexes is depicted in Figure 1. Cr(CO)_6_ is a complex with 18 valence electrons (VEs) and adopts an octahedral molecular geometry, resulting in the *O_h_* point group symmetry. Similarly, (η^6^-C_6_H_6_)Cr(CO)_3_ has 18 VEs. However, it has a piano stool geometry with planar arrangement of the aryl group and three carbonyl groups which act as “legs”. Both complexes have spin-paired electrons and accordingly are diamagnetic. For such compounds, it has been postulated that DEA leads exclusively to the formation of fragment negative ions [26], without formation of parent anions, since the captured electron has to occupy an antibonding molecular orbital. In accordance with the predictions, we have observed the formation of three and seven fragment anions from Cr(CO)_6_ and (η^6^-C_6_H_6_)Cr(CO)_3_, respectively, while the parent anion was not observed from any of the investigated complexes. In the following, the present results will be discussed in this context and in relation to the role of ligands in the respective DEA processes and compared with available literature data.

## Experimental

Experiments were performed utilizing an electron-molecular crossed beam setup. As previously described in [27], it consists of a trochoidal electron monochromator (TEM), a quadrupole mass analyzer (QMA), and a secondary electron multiplier, which are housed in a high vacuum chamber. The electron beam (energy resolution in the range of 150–200 meV (FWHM), electron current *I* ≈ 10 nA) generated with the TEM intersects with an effusive molecular beam, resulting in the formation of fragment anions. The molecular beam emanates from a vessel which was introduced directly into the oven in the vacuum chamber. In order to obtain sufficiently high vapor pressure of the target molecules in the reaction area, the oven was heated by two halogen lamps to a temperature of 90–95 °C as measured by a Pt(100) resistance mounted at one of the flanges. The generated negative ions were extracted from the reaction area by a small electric field towards the QMA entrance and detected by a single pulse counting technique. The electron energy scale was calibrated by measuring the signal of SF_6_^−^, exhibiting an intense resonance near 0 eV. Base pressure was in the range of 3 × 10^−8^ mbar while the working pressure was in the range of either 4–5 × 10^−7^ mbar or 2–4 × 10^−5^ mbar for Cr(CO)_6_ and (η^6^-C_6_H_6_)Cr(CO)_3_, respectively. In spite of almost the same operative temperature for both investigated compounds, the pressure of the homoleptic compound was substantially lower in comparison with the heteroleptic compound. This implies a lower sublimation rate for the former compound. The Cr(CO)_6_ and (η^6^-C_6_H_6_)Cr(CO)_3_ samples were purchased from Sigma-Aldrich with a stated purity of 98% and used as-delivered.

## Results and Discussion

The impact of low-energy electrons on gas-phase chromium(0) hexacarbonyl (Cr(CO)_6_) and benzene-chromium(0) tricarbonyl ((η^6^-C_6_H_6_)Cr(CO)_3_) has been investigated. Measurements have been taken as a function of incident electron energy in the energy range between 0–12 eV. In this energy range, it is very well known that DEA is responsible for the dissociation of the molecule. The DEA reaction is a two-step process in which, in a first step, an incident electron is captured by the target molecule to form a transient negative ion (TNI). Since the TNI is not stable, it will decay in a second step either via autoionisation or via dissociation, forming a stable fragment anion and neutral counterpart(s). The formation of the fragment anion is only possible if the fragment at which the extra charge is localized possesses a positive value of the electron affinity. Depending on the energy at which DEA occurs, one can distinguish two types of resonances: one-particle resonance and two-particle one-hole resonance (core excited resonance) [28]. One-particle resonances take place within the subexcitation energy range below 3–4 eV. They are due to a direct accommodation of the excess electrons into an unoccupied molecular orbital (MO). On the other hand, core excited resonances occur when the incoming electron transfers its energy to electronically excite the target molecule and hence becomes captured by the electron–molecule potential of the excited state.

**Figure 2:**
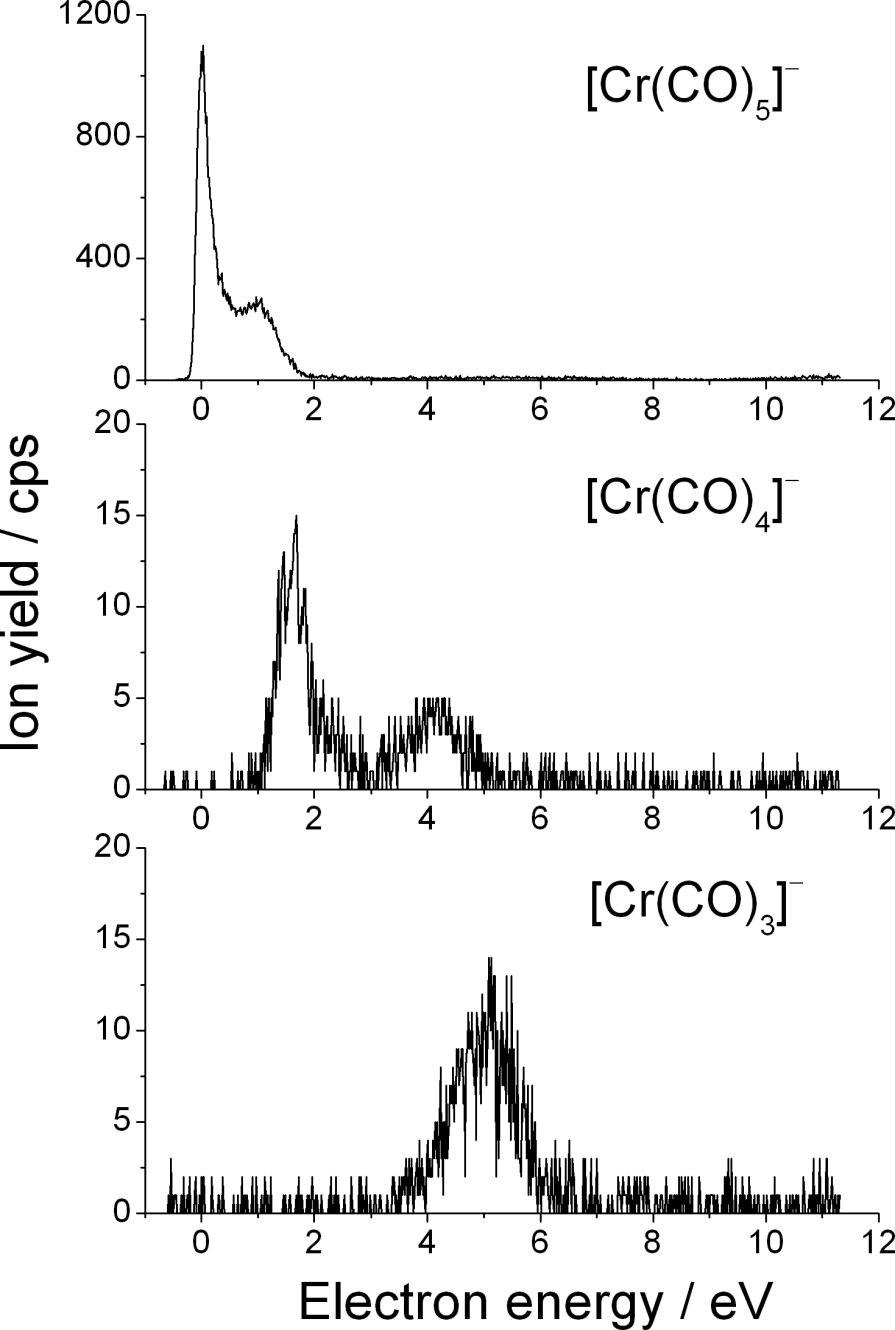
Yield of the fragment anions generated from DEA to chromium(0) hexacarbonyl.

Electron attachment to Cr(CO)_6_ leads, under the current experimental conditions, to the formation of three anionic fragments, [Cr(CO)_5_]^−^, [Cr(CO)_4_]^−^, and [Cr(CO)_3_]^−^. The ion yield curves of these fragments are shown in Figure 2. The corresponding fragment anions are generated via:


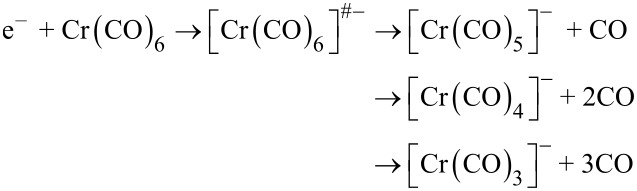


The predominant reaction channel is the formation of [Cr(CO)_5_]^−^ through the cleavage of one CO ligand from the transient negative ion. This fragment shows a narrow and intense structure close to 0 eV within the ion yield curve and a less intense peak at 1 eV, which are attributed to one-particle resonances. The positions of the peaks correlate very well with the positions of the resonances reported from electron transmission spectroscopy (ETS) experiments [29]. Indeed, from ETS, the features emerge below 1 eV implying the occurrence of an attachment of electrons with energy close to 0 eV and near 1 eV. Based on the calculations, it has been suggested that the low energy maximum can be attributed to a negative ion state emerging from electron capture into the 3t_2g_ orbital of this molecule [30]. A high cross section for the [Cr(CO)_5_]^−^ ion formation already at energy close to 0 eV implies that the reaction is most likely exothermic, i.e., the electron affinity of the Cr(CO)_5_ fragment exceeds the bond energy of Cr–CO. According to the literature, the bond dissociation enthalpy of Cr(CO)_5_–CO is equal to 1.6 eV [31], therefore the electron affinity of Cr(CO)_5_ should be ≥1.6 eV. A further reaction channel in DEA to Cr(CO)_6_ is the formation of [Cr(CO)_4_]^−^, formed from the loss of two CO ligands. The ion yield curve is composed of two peaks, centered at 1.4 eV and 4.1 eV. The position of the first peak correlates well with the position of the resonance observed from ETS studies that has been assigned to capture into the 3t_2u_ orbital of this molecule [30]. Further abstraction of the CO ligand leads to the formation of [Cr(CO)_3_]^−^ which extends from 3.3 to 6 eV and peaks at 5.0 eV. This structure can be attributed to a two-particle one-hole resonance. However, it should be stressed here that one-particle resonances may also be formed in this energy range and cannot be ruled out.

DEA studies on Cr(CO)_6_ have already been reported by Tossell et al. [30] as well as Winters and Kiser [32]. In the former paper, the negative ion current from Cr(CO)_6_ as a function of incident electron energy has been shown. The reported dominant feature was observed near 0.5 eV with shoulders between 1 and 2 eV and between 2 and 3 eV, which is in a fairly good agreement with our experimental results. The authors concluded that the vast majority of the ions observed were [Cr(CO)_5_]^−^. The only exception was a peak near 1.6 eV where [Cr(CO)_4_]^−^ contributed about 15% to the total ion current. In contrast, Winters and Kiser have observed formation of six fragment anions, i.e., [Cr(CO)_5_]^−^, [Cr(CO)_4_]^−^, [Cr(CO)_3_]^−^, [Cr(CO)_2_]^−^, [Cr(CO)]^−^, and [Cr]^−^ [32]. In analogy to our results, the most intense fragment was generated from the cleavage of a single CO ligand. The [Cr(CO)_4_]^−^ and [Cr(CO)_3_]^−^ anions were generated with an intensity of 30% of the main signal. Further signals were observed with relative intensity of 10%, 5%, and <5% for [Cr(CO)_2_]^−^, [Cr(CO)]^−^, and [Cr]^−^, respectively. Hence, the intensity of the missing fragments in our experiment is below the detection limit of our experimental setup. In particular, if we take into account that the intensity of the [Cr]^−^ signal in the experiment of Winters and Kiser is lower than 5% of [Cr(CO)_5_]^−^ at 2.4 eV, we end up at the noise level. However, in their studies, Winters and Kiser could only observe a single peak for any of the reported fragment anions appearing above 2 eV. It should be stressed here that the maximum of the second peak for [Cr(CO)_4_]^−^ and the maximum of the peak for [Cr(CO)_3_]^−^ observed within the present studies match reasonably well to the position of the peaks reported by Winters and Kiser. There is, however, a big discrepancy between the positions of the dominant [Cr(CO)_5_]^−^ fragment. While in our studies the main peak appears already near 0 eV with a contribution at 1 eV, from the experiment of Winters and Kiser, it is reported to peak at 2.4 eV. It should be emphasized that the appearance of all observed anions, including the main peak of the [Cr(CO)_5_]^−^ ion, above 2 eV in the latter studies contradicts also with the results of George and Beauchamp, who have shown that the rate constant at the thermal energy for dissociative electron capture by Cr(CO)_6_ approaches the maximum thermal attachment rate constant and equals to 3.2 × 10^−7^ cm^3^·molecule^−1^·s^−1^ [13].

A particularly extensive fragmentation has been observed in DEA to the heteroleptic complex (η^6^-C_6_H_6_)Cr(CO)_3_. In the case of electron attachment to (η^6^-C_6_H_6_)Cr(CO)_3_, we observe the production of seven fragment anions: [(C_6_H_6_)Cr(CO)_2_]^−^, [(C_6_H_6_)Cr(CO)]^−^, [(C_6_H_6_)Cr]^−^, [Cr(CO)_3_]^−^, [Cr(CO)_2_]^−^, [CrCO]^−^, and Cr^−^ in the electron energy range of 0–12 eV. These fragments can be divided into two groups: a first group which contains the anions generated by the loss of one or more CO ligands and the second one which includes the anions formed from the loss of the C_6_H_6_ unit or the loss of C_6_H_6_ and one or more CO units. The fragment anions which form the first group are generated via:


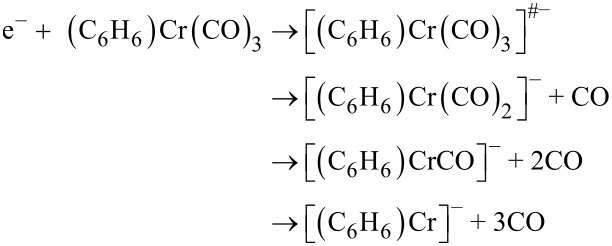


**Figure 3:**
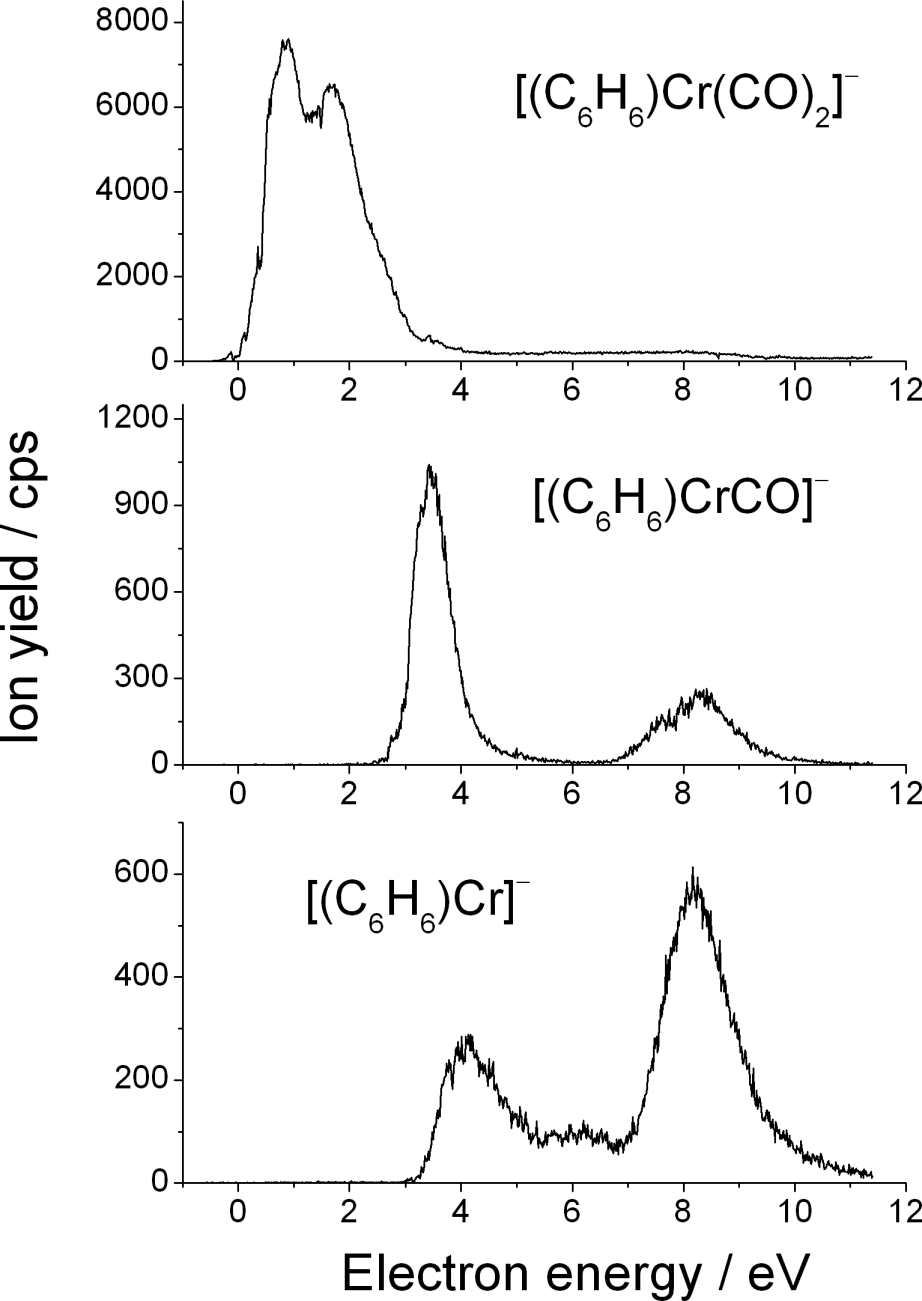
Yield of the fragment anions [(C_6_H_6_)Cr(CO)_2_]^−^, [(C_6_H_6_)Cr(CO)]^−^, and [(C_6_H_6_)Cr]^−^ obtained from DEA to benzene-chromium(0) tricarbonyl which are generated by the loss of one, two, and three carbonyl groups, respectively.

The ion yield curves of these fragments are shown in Figure 3. At first glance it becomes obvious that the predominant fragment is due to the loss of one CO ligand. This is consistent with our results obtained for the homoleptic chromium complex Cr(CO)_6_ as well as the previously studied ruthenium complex containing multicoordinated π-allyl ligands (η^3^-C_3_H_5_)Ru(CO)_3_Br [19]. The [(C_6_H_6_)Cr(CO)_2_]^–^ anion is visible via two strongly overlapping structures at 0.85 eV and 1.7 eV. Hence, it is shifted towards higher energy with respect to the position of the fragment anion formed from the loss of a single CO ligand from Cr(CO)_6_. This shift can be caused mainly by the fact that chromium–carbonyl bonds are stronger in the heteroleptic complex (η^6^-C_6_H_6_)Cr(CO)_3_ than in homoleptic Cr(CO)_6_ (see [31] and references therein). The [(C_6_H_6_)CrCO]^–^ channel associated with the loss of two CO groups has a threshold at 2.5 eV and two resonances are present within the yield curve peaking at 3.5 eV and 8.3 eV. The abstraction of a further CO unit results in the formation of [(C_6_H_6_)Cr]^−^. The threshold for this reaction channel is somewhat shifted towards higher energy, that is to 3.3 eV, in comparison to the fragment formed from the loss of two CO units. Within the ion yield curve one can distinguish three resonances centered at 4.1, 6.0 and 8.2 eV. From Figure 3 it can be clearly seen that the efficiency of the reaction channel decreases with the number of carbonyl groups that are detached from the TNI to form the respective anions. This behavior has already been reported from DEA to metal carbonyls (e.g., Ni(CO)_4_, Fe(CO)_5_, Cr(CO)_6_, Mo(CO)_6_, W(CO)_6_) [32], as well as from cobalt tricarbonylnitrosyl [9], and π-allyl ruthenium tricarbonyl bromide [19].

The second group of anionic products is formed by the following four dissociative channels:


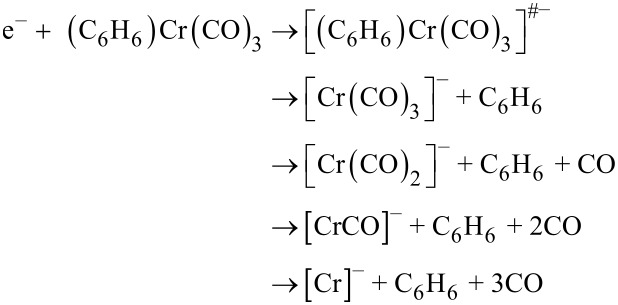


**Figure 4:**
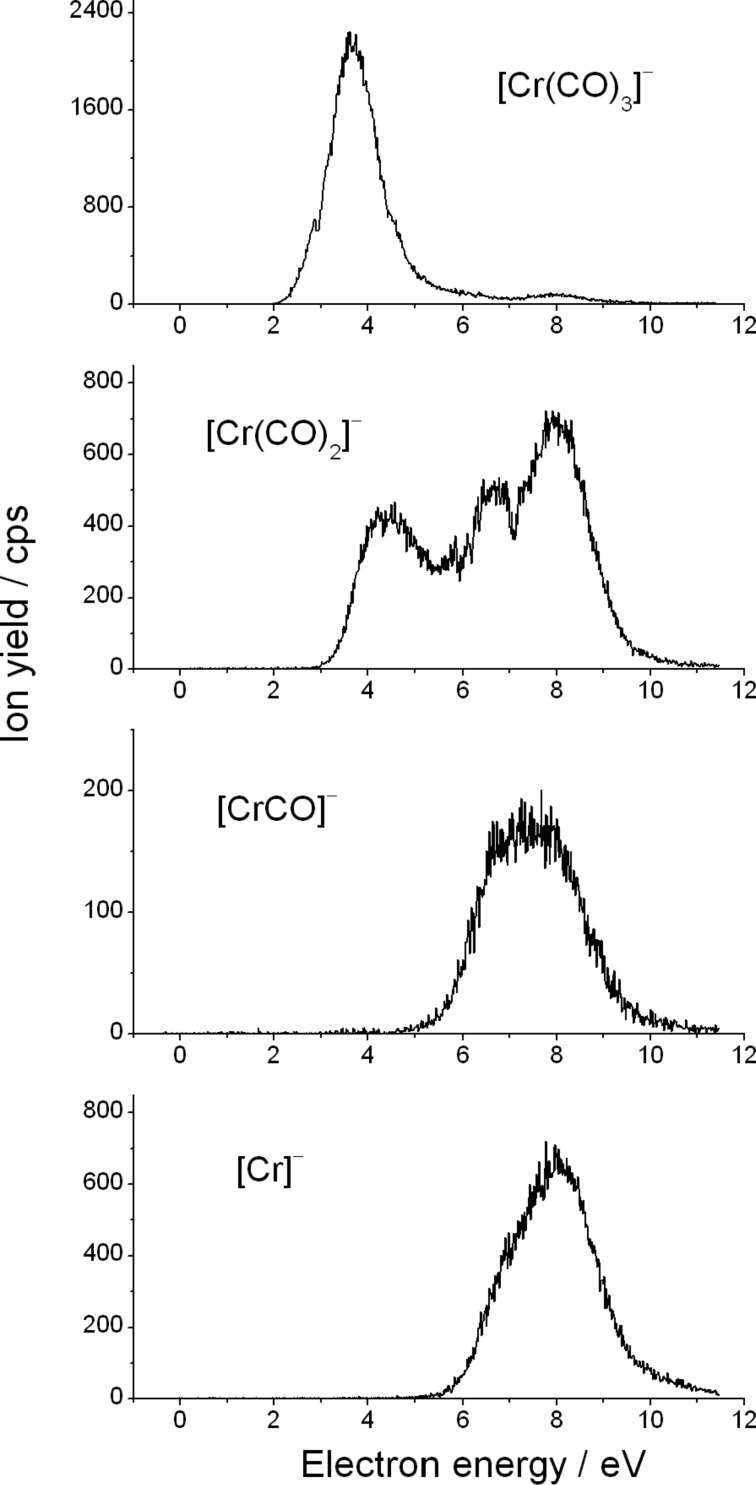
Yield of the fragment anions [Cr(CO)_3_]^−^, [Cr(CO)_2_]^−^, [CrCO]^−^, and Cr^−^ obtained from DEA to benzene-chromium(0) tricarbonyl.

As mentioned above, this group consists of fragment anions generated from the loss of the C_6_H_6_ ligand or the loss of C_6_H_6_ and one or more CO ligands to form [Cr(CO)_3_]^−^, [Cr(CO)_2_]^−^, [CrCO]^−^, and Cr^−^, respectively (Figure 4). The predominant fragment from the second group is formed as a result of the loss of the C_6_H_6_ unit. The main contribution to the ion yield of [Cr(CO)_3_]^−^ is visible through a resonance structure peaking at 3.7 eV with a low intensity structure at around 7.9 eV. Considering all the fragments generated from DEA to (C_6_H_6_)Cr(CO)_3,_ this is the second most intense signal. Such a high intensity of [Cr(CO)_3_]^−^ has to be related to the lability of the C_6_H_6_ group. This is a striking finding since, in general, the multicentered π-bonded ligands are recognized to be particularly persistent in FEBID, and hence should be avoided [18–19]. However, it should be emphasized here that the C_6_H_6_ group is neutral in contrast to, for example, the methylcyclopentadienyl ligand, which may facilitate the detachment of the C_6_H_6_ group from the (C_6_H_6_)Cr(CO)_3_ complex.

The [Cr(CO)_2_]^−^ anion extends in a very broad energy range from 3.3 eV to 10 eV and is visible via three strongly overlapping resonant structures peaking at 4.5, 6.5 and 7.9 eV. Further loss of CO results in the formation of [CrCO]^–^ which appears in the high energy domain with a peak maximum at 7.5 eV. Since the peak is very broad, the threshold for the reaction channel is as low as 5.5 eV. Finally, we would like to point out that we observe the formation of a bare chromium anion, Cr^−^. The yield curve of Cr^−^ stretches from 6 to 11 eV and peaks around 8 eV and has a pronounced shoulder at the low energy side. It is worth mentioning that Cr^−^ is generated with an exceptionally high relative cross section, which is untypical when considering organometallic complexes including those with multicoordinated ligands. Indeed, as obvious from the reports on (η^3^-C_3_H_5_)Ru(CO)_3_Br [19], MeCpPtMe_3_[18], Co(CO)_3_NO [9], and HFeCo_3_(CO)_12_ [33], the bare metal ions were either not observed or observed with very low intensity (below 0.5% of the most intense anionic fragment).

## Conclusion

In the present contribution, we have investigated how different ligands within coordination complexes modify the formation of transient negative ions (TNIs) and their subsequent decay by dissociation. In particular, by selecting homo- and heteroleptic complexes, namely Cr(CO)_6_ and (η^6^-C_6_H_6_)Cr(CO)_3_, we have studied the influence of the substitution of three CO units by the hexahapto C_6_H_6_ unit on electron-induced fragmentation. In general, we have observed the electron-driven decomposition of chromium(0) hexacarbonyl and benzene-chromium(0) tricarbonyl complexes into three ([Cr(CO)_5_]^−^, [Cr(CO)_4_]^−^, and [Cr(CO)_3_]^−^) and seven ([(C_6_H_6_)Cr(CO)_2_]^−^, [(C_6_H_6_)Cr(CO)]^−^, [(C_6_H_6_)Cr]^−^, [Cr(CO)_3_]^−^, [Cr(CO)_2_]^−^, [CrCO]^−^, and Cr^−^) fragment anions, respectively. The energy of the peak maxima and intensity of the [M–(CO)*_x_*]^−^ fragment anions (where M = neutral molecule, and *x* can be equal to 1, 2 or 3) generated from both investigated complexes varied in an anticipated fashion. Such a stepwise increase in the energy and decrease in the intensity as more CO ligands are removed has been previously reported for a series of transition-metal carbonyls [14,32]. It has also been suggested that such a behavior may be reminiscent of a successive removal of CO fragments in the cracking patterns of the negative ions. Hence, it is very likely that the [M(CO)*_x_*]^−^ anions, besides the direct decomposition of TNI, may also be generated via sequential, metastable decay.

In the framework of the potential role of coordination complexes of chromium for FEBID applications, we note that the removal of C_6_H_6_ and all CO ligands to form exceptionally intense signal of bare [Cr]^−^ has been observed in DEA to (η^6^-C_6_H_6_)Cr(CO)_3_. On the basis of our results it seems to be plausible to consider a multicentered benzyl group as a promising leaving group within FEBID precursors.
